# Strongly Anchoring Polysulfides by Hierarchical Fe_3_O_4_/C_3_N_4_ Nanostructures for Advanced Lithium–Sulfur Batteries

**DOI:** 10.1007/s40820-020-00475-5

**Published:** 2020-07-01

**Authors:** Soochan Kim, Simindokht Shirvani-Arani, Sungsik Choi, Misuk Cho, Youngkwan Lee

**Affiliations:** 1 School of Chemical Engineering, Sungkyunkwan University, Suwon, 16419 Republic of Korea; 2 Nuclear Science and Technology Research Institute (NSTRI), Tehran, 14395-834 Iran

**Keywords:** Hierarchical nanostructured C_3_N_4_, Fe_3_O_4_ nanosphere, Interlayer, Long-term cycling, Lithium–sulfur battery

## Abstract

Li–S batteries have attracted considerable interest as next-generation energy storage devices owing to high energy density and the natural abundance of sulfur. However, the practical applications of Li–S batteries are hampered by the shuttle effect of soluble lithium polysulfides (LPS), which results in low cycle stability. Herein, a functional interlayer has been developed to efficiently regulate the LPS and enhance the sulfur utilization using hierarchical nanostructure of C_3_N_4_ (*t*-C_3_N_4_) embedded with Fe_3_O_4_ nanospheres. *t*-C_3_N_4_ exhibits high surface area and strong anchoring of LPS, and the Fe_3_O_4_/*t*-C_3_N_4_ accelerates the anchoring of LPS and improves the electronic pathways. The combination of these materials leads to remarkable battery performance with 400% improvement in a specific capacity and a low capacity decay per cycle of 0.02% at 2 C over 1000 cycles, and stable cycling at 6.4 mg cm^−2^ for high-sulfur-loading cathode.

**Electronic supplementary material:**

The online version of this article (10.1007/s40820-020-00475-5) contains supplementary material, which is available to authorized users.

## Highlights

- A multi-functional interlayer for Li–S batteries has been developed to efficiently regulate the shuttle effect and enhance the sulfur utilization using hierarchical Fe_3_O_4_/C_3_N_4_ nanostructures.
- C_3_N_4_ nanotube exhibits high surface area and high affinity toward LPS, and the decorating Fe_3_O_4_ nanospheres on the surface of C_3_N_4_ nanotube accelerate the adsorption of LPS and improve the electronic pathways.
- The combination of Fe_3_O_4_ nanospheres and C_3_N_4_ nanotube leads to remarkable battery performance with 400% improvements in specific capacity compared with Li–S battery without using interlayer and a low capacity decay per cycle of 0.02% at 2 C over 1000 cycles.

**Electronic supplementary material:**

The online version of this article (10.1007/s40820-020-00475-5) contains supplementary material, which is available to authorized users.

## Graphical abstract

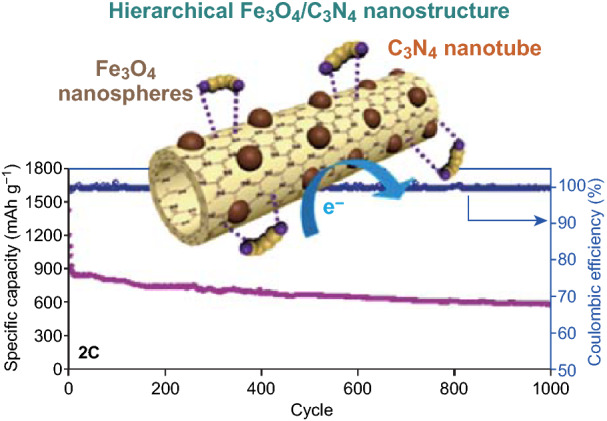

## Introduction

Rechargeable lithium–sulfur batteries (LSBs) are widely expected to be the next-generation high-density energy storage technology due to their high theoretical capacity and high energy density, as well as the natural abundance and environmental compatibility of sulfur [1, 2]. However, the practical applications of LSBs are still hampered by intrinsic issues, such as the large volumetric expansion (80%) of sulfur upon lithiation, insulating properties of sulfur, and dissolution of intermediate lithium polysulfides (LPS) species during cycling [1–3]. Shuttle effects, which are a result of the elution of LPS, are considered to be the main issue, resulting in the low Coulombic efficiency, high self-discharge, poor rate performance, and low charge–discharge cycles of LSBs [3, 4]. To address this main issue, several strategies to regulate the LPS have been explored by various research groups. The most studies are focused on the control of the LPS generated from the sulfur cathode, such as the encapsulation of sulfur and the development of functional polymer binders for LSB. These approaches have led to the improved suppression of the LPS shuttle effect, but they do not control the LPS generated after long-term cycling and the resulting LPS passivate the Li-metal surface [5, 6].

Recently, an interlayer has been introduced for the effective adsorption of LPS and for the reuse of the adsorbed active material on the outside of the cathode. Various carbon materials have been used as interlayers. To improve their LPS adsorption properties, N, O, or S doping, combination with various carbon materials, and composites with metal compounds are introduced [7–11].

In particular, N-doped carbon materials effectively inhibit the LPS shuttle effect and lead to prolonged battery cycling due to the strong chemical interactions formed between the electronegative nitrogen atoms and the Li ions in the LPS [12–14]. However, the introduction of N-doped graphene and N-doped carbon nanotubes complicates the manufacturing process and is expensive [15, 16]. In contrast, graphitic carbon nitride (g-C_3_N_4_), which contains tri-s-triazine units connected to planar amino groups in each layer, is a promising functional material owing to its inexpensive precursor, easy manufacturing process, and high nitrogen content (> 60%) [16, 17]. However, the limited surface area and relatively low electrical conductivity of g-C_3_N_4_ are significant challenges to be solved because they can cause inadequate battery performance.

In this work, we have introduced a hierarchical nanostructured C_3_N_4_-based material for use as an interlayer with improved electrical conductivity and efficient anchoring LPS. For the hierarchical nanostructures, tubular C_3_N_4_ (*t*-C_3_N_4_) is prepared from cheap melamine without template and Fe_3_O_4_ nanospheres are decorated on the surface of *t*-C_3_N_4_ by facile synthetic method. Tubular morphology of C_3_N_4_ exhibits a high surface area, which results in an increase in its contact with the LPS. Fe_3_O_4_ nanoparticles are known as having high interaction with LPS, excellent electrical conductivity, and low cost among various metal oxides [18]. The as-developed hierarchical tubular C_3_N_4_ (*t*-C_3_N_4_) embedded with Fe_3_O_4_ nanospheres (Fe_3_O_4_/*t*-C_3_N_4_) has been applied as an interlayer and shows remarkable battery performance with 400% improvements in specific capacity compared with LSB without using interlayer and a low capacity decay per cycle of 0.02% at 2 C over 1000 cycles. Moreover, it presented the stable cycling at 6.4 mg cm^−2^ for high-sulfur-loading cathode. With facile synthesis, low-cost precursors, and low toxic materials, optimally designed Fe_3_O_4_/*t*-C_3_N_4_ for sulfur cathode would be a new approach for high-performance energy storage devices.

## Experimental

### Materials

Melamine, FeSO_4_·7H_2_O, FeCl_3_·6H_2_O, NaOH, sulfur, lithium metal, bis(trifluoromethyl sulfonyl)amine lithium salt (LiTFSI), 1,3-dioxolane (DOL), dimethoxyethane (DME), and poly(vinylidene fluoride) (PVdF) (M_w_ 530,000 g mol^−1^) were purchased from Sigma-Aldrich (USA). Lithium nitrate (LiNO_3_), Li_2_S, and *N*-methyl-2-pyrrolidinone (NMP) were purchased from Alfa Aesar (USA).

### Synthesis of g-C_3_N_4_ and t-C_3_N_4_

g-C_3_N_4_ was prepared via thermal polymerization using melamine as a precursor. Melamine powder (3 g) was heated in air to 550 °C at a heating rate of 10 °C min^−1^. The g-C_3_N_4_ product was obtained after being heated at 550 °C for 5 h and naturally cooled to room temperature. *t*-C_3_N_4_ was obtained via a two-step process based on rolling-up mechanism (detailed in electronic supplementary material). Firstly, melamine (3 g) was added to 30 mL of deionized water and dispersed for 30 min using tip sonication (750 W, VCX 750, Sonics, USA). The resulting mixture was heated in a Teflon-lined autoclave at 200 °C for 12 h. After the autoclave was cooled to room temperature, a white powder was obtained. Following filtration and washing with deionized water and ethanol for three times, the white powder was ground in an agate mortar for 30 min. The ground white powder was heated under above-mentioned conditions to give the *t*-C_3_N_4_ product (heated in air to 550 °C at a heating rate of 10 °C min^−1^ and 550 °C for 5 h).

### Synthesis of Hierarchical Nanostructured C_3_N_4_ Embedded with Fe_3_O_4_ Nanospheres (Fe_3_O_4_/t-C_3_N_4_)

A specific amount of *t*-C_3_N_4_ was dispersed in ethanol/H_2_O (1:1) using ultrasonication for 2 h. An aqueous solution containing FeSO_4_·7H_2_O (1.313 mmol) and FeCl_3_·6H_2_O (1.85 mmol) was prepared and specific aliquots of the resulting solution were then added to the *t*-C_3_N_4_-containing suspension under vigorous stirring at 80 °C. After 10 min, 2 M NaOH solution was rapidly added to the above-mentioned solution to reach a pH of ~ 10. After stirring for 30 min at 80 °C, the resulting black mixture was allowed to cool to room temperature. The mixture was washed several times with H_2_O and ethanol and then dried in an oven at 60 °C for 12 h to obtain the Fe_3_O_4_-decorated *t*-C_3_N_4_ product. According to the amount of *t*-C_3_N_4_ used, L-Fe_3_O_4_/*t*-C_3_N_4_ (low concentration of Fe_3_O_4_), Fe_3_O_4_/*t*-C_3_N_4_, and H-Fe_3_O_4_/*t*-C_3_N_4_ (high concentration of Fe_3_O_4_) were obtained.

### Preparation of Interlayer

The interlayer was obtained by coating the as-synthesized material on a Celgard (2500) membrane. g-C_3_N_4_, *t*-C_3_N_4_ or Fe_3_O_4_/*t*-C_3_N_4_, carbon black (CB), and 5 wt % PVdF (in NMP) were mixed in a weight ratio of 6/3/1 (as-synthesized materials/CB/binder, *w*/*w*/*w*) using high-energy ball milling (8000D, SPEX, USA). The mixed slurry was then homogeneously coated onto the Celgard (2500) membrane using a doctor blade. The interlayer was dried at 50 °C under an air atmosphere for 12 h and at 60 °C in vacuum for 3 h. The typical loading was 0.3–0.5 g cm^−2^.

### Characterization

The morphology and EDS analysis of the as-prepared materials (g-C_3_N_4_, *t*-C_3_N_4_, and Fe_3_O_4_/*t*-C_3_N_4_) were obtained using transmission electron microscopy (TEM, JEM-2010, JEOL, Japan). The surface morphology of the interlayer was observed using scanning electron microscopy (SEM, JSM-6390A and JSM-7500F, JEOL, Japan). X-ray diffraction (XRD) patterns were collected using a D8 Advance diffractometer (Bruker, Germany) equipped with a Cu Kα radiation source (λ = 1.54056 Å). X-ray photoelectron spectroscopy (XPS) was performed using an ESCA 2000 spectrometer (VG Scientific, USA) with an Al-Kα X-ray source operated at 170 W (13 mA and 13 kV). Raman spectra were obtained to characterize Fe_3_O_4_/*t*-C_3_N_4_ at room temperature (RT) using Raman spectrometry (DXR2xi, Thermo Fisher, USA) with a 785-nm laser excitation source. Elemental analysis (EA) was conducted using a Vario EL cube (Elementar, Germany). Thermogravimetric analysis (TGA) was performed from 25 to 800 °C at a heating rate of 10 °C min^−1^ under a flow of N_2_ using a Seiko Exstar 6000 TG/DTA6100 instrument (Seiko, Japan). The specific surface area (BET) was determined on a surface area analyzer (ASAP 2020 Plus, Micromeritics Instruments, USA) using the Brunauer–Emmett–Teller method. Electrical impedance spectroscopy (EIS) was performed on a potentiostat (VSP, BioLogic, France). The LPS adsorption capability was evaluated using UV–Vis spectroscopy (8453 UV–visible Spectroscopy System, Agilent, USA) and the LPS adsorption capability of prepared interlayer was observed using H-type cells.

### Electrochemical Characterization

Electrochemical characterization was carried out using CR2032-type coin cells. To prepare the sulfur cathode, the sulfur/CB composite (7/3, *w/w*), CB, and 5 wt% PVdF (in NMP) were mixed in a weight ratio of 8:1:1 (sulfur composites/CB/binder, *w*/*w*/*w*) using high-energy ball milling. The mixed slurry was then homogeneously coated onto aluminum foil (20 μm, thickness) via a doctor blade. Finally, the electrodes were dried at 50 °C under an air atmosphere for 12 h and at 50 °C in vacuum for 6 h. The typical sulfur loading was 0.8–1.0 mg cm^−2^. In the case of a high-sulfur loading, the cathodes were fabricated on carbon paper (AvCarb, Ballard, USA) using a similar procedure to that described above. The electrolyte was a mixture of DOL and DME (1:1, *v/v*) containing 1 M LiTFSI and 0.2 M LiNO_3_ and added to Li–S cell with a ratio of 20 μL/mg-S. The Li–S cells were assembled in a 2032-type coin cell using the sulfur cathode as the working electrode, lithium metal as the counter electrode, and the Celgard membrane (2500) or fabricated interlayer as the separator in an Ar-filled glove box containing < 10 ppm H_2_O and O_2_. The galvanostatic discharge–charge behavior was monitored using a battery test system (WonATech Corp., Republic of Korea) over a voltage range of 1.7–2.8 V vs. Li/Li^+^ at 25 °C and all the Li–S cells were activated at 0.05 C for two cycles prior to measurement. EIS measurements were conducted using a potentiostat (VSP, BioLogic, France) upon applying a 50-mV-amplitude sine wave in the frequency range of 0.1 Hz to 100 kHz.

## Results and Discussion

### Characterization of Fe_3_O_4_/*t*-C_3_N_4_

**Fig. 1:**
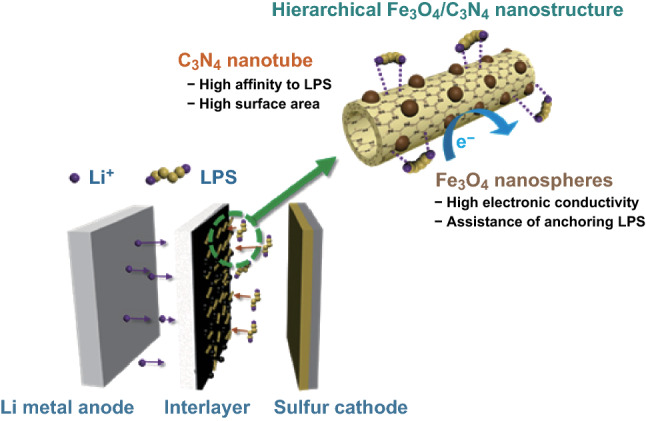
A schematic representation of the roles of the Fe_3_O_4_/*t*-C_3_N_4_ interlayer

Figure 1 illustrates the roles of the Fe_3_O_4_/*t*-C_3_N_4_ interlayer in the effective anchoring LPS, preventing dissolved LPS from traveling to the lithium metal anode during the discharge process. *t*-C_3_N_4_ exhibits a high affinity for LPS and high surface area, and the decorated Fe_3_O_4_ nanospheres on *t*-C_3_N_4_ provide high electronic conductivity and contribute additional LPS adsorption and sulfur utilization.

**Fig. 2:**
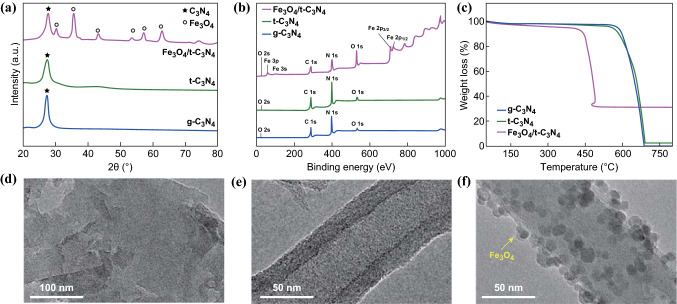
a XRD patterns, **b** XPS spectra, and **c** TGA curves obtained for g-C_3_N_4_, *t*-C_3_N_4_, and Fe_3_O_4_/*t*-C_3_N_4_. TEM images recorded for **d** g-C_3_N_4_, **e** *t*-C_3_N_4_, and **f** Fe_3_O_4_/*t*-C_3_N_4_

XRD, XPS, TGA, and TEM were used to confirm the structure of the as-prepared materials (g-C_3_N_4_, *t*-C_3_N_4_, and Fe_3_O_4_/*t*-C_3_N_4_), as shown in Fig. 2. All of the as-prepared materials exhibit a specific peak corresponding to g-C_3_N_4_, which suggests that the samples have the same basic crystal structure of g-C_3_N_4_ (JCPDS No. 87-1526). The strong inter-planar stacking peak of g-C_3_N_4_ observed at 27.2° (002), which corresponds to the aromatic systems, was shifted to 27.5° in *t*-C_3_N_4_, indicating the decreased gallery distance between the basic sheets of *t*-C_3_N_4_ [19, 20]. In the case of Fe_3_O_4_/*t*-C_3_N_4_, the low-angle reflection peak was shifted to 27.7°, which was mainly attributed to the simultaneous decrease in the planar size of the layers and the formation of Fe_3_O_4_ nanospheres. Considering the characteristic peaks of Fe_3_O_4_ (JCPDS No. 19-0629), the presence of Fe_3_O_4_ nanospheres on *t*-C_3_N_4_ does not influence the crystal structure of *t*-C_3_N_4_, which is advantageous for its affinity toward LPS and enhancement of electronic conductivity by sharing their respective effects.

XPS was employed to investigate the valence states and chemical environment of the constituent elements on the surface of the g-C_3_N_4_, *t*-C_3_N_4_, and Fe_3_O_4_/*t*-C_3_N_4_ materials (Figs. 2b and S1). Both g-C_3_N_4_ and *t*-C_3_N_4_ display similar N 1 s spectra, which confirm the existence of a graphite-like *sp*^2^-bonded structure. Moreover, two peaks were observed at 725 and 711 eV in the Fe 2p spectrum recorded for the Fe_3_O_4_/*t*-C_3_N_4_, which were assigned to Fe 2p_1/2_ and Fe 2p_3/2_, respectively (Fig. S1a). Detailed N 1 s peaks are shown in Fig. S1b. The values are almost equal to the standard binding energy of Fe_3_O_4_, suggesting Fe_3_O_4_ deposited on the region of *t*-C_3_N_4_ [21, 22]. Moreover, the Fe_3_O_4_/*t*-C_3_N_4_ is characterized by Raman spectroscopy (detailed in Fig. S2).

To confirm the thermal properties of the as-prepared materials, TGA was performed under a flow of N_2_ from 25 to 800 °C (Fig. 2c). g-C_3_N_4_ and *t*-C_3_N_4_ are stable below 550 °C. When the temperature increases above 550 °C, the decomposition of C_3_N_4_ occurs and is completed at 700 °C. For Fe_3_O_4_/*t*-C_3_N_4_, the TGA curve was shifted to lower temperature due to the catalytic effects of Fe_3_O_4_ nanospheres on the oxidation of *t*-C_3_N_4_ [23]. Using the residual weight observed at > 440 °C, the Fe_3_O_4_ content was determined to be ~ 30 wt%. Moreover, through elemental analysis (EA, CHN mode), composition of g-C_3_N_4_ and *t*-C_3_N_4_ was further confirmed (Table S2). In the present investigation, the theoretical value of C/N atomic ratio is 0.75. The C/N ratios in the cases of g-C_3_N_4_ and *t*-C_3_N_4_ are found to be a similar result of theoretical value (0.66 and 0.69, respectively).

TEM analysis was carried out to characterize the structure of the as-prepared materials. The TEM images in Fig. 2d–f show that the tubular structure of *t*-C_3_N_4_ was well formed with diameters of ~ 50 nm when compared to the shee*t*-like morphology of g-C_3_N_4_. Moreover, Fe_3_O_4_ nanospheres with diameter of 2–5 nm were found to be dispersed on the surface of *t*-C_3_N_4_ (Fig. 2f). Moreover, EDS analysis was conducted and confirmed the existence of C, N, O, and Fe elements in Fe_3_O_4_/*t*-C_3_N_4_ (Fig. S3). Consequently, the well-dispersed Fe_3_O_4_ nanospheres on *t*-C_3_N_4_ will enhance electronic conductivity and provide additional LPS adsorption.

To evaluate the electronic conductivity of prepared materials, we assembled the cell (prepared materials coated cathode, separator, Li-metal anode, and electrolyte). In Fig. S4, the cell with Fe_3_O_4_/*t*-C_3_N_4_ material presents a smaller resistance (*R*_ct_, see the electronic supplementary material for details) when compared to those of the as-prepared other cells. This was attributed to enhanced electronic conductivity from decorated Fe_3_O_4_ on *t*-C_3_N_4_, and the data suggest that Fe_3_O_4_/*t*-C_3_N_4_ can provide fast electronic transfer and enhance an efficient electrochemical system [4, 5, 8].

The morphological changes depending on the Fe_3_O_4_ loading amount are shown in Fig. S5. Using a low concentration of the Fe-based precursor, only a few Fe_3_O_4_ nanospheres were sparsely attached on the surface of *t*-C_3_N_4_, whereas the Fe_3_O_4_ nanospheres were aggregated on the surface of *t*-C_3_N_4_ when using a high concentration of the Fe precursor. According to Liao et al. and Ding et al., the binding energy between C_3_N_4_ and Li_2_S_6_ is 4.37 times higher than that between Fe_3_O_4_ and Li_2_S_6_ from density functional theory computational calculations [24, 25]. Therefore, a dense Fe_3_O_4_ nanosphere surface may limit the chemical interaction of *t*-C_3_N_4_ with the LPS. The deposition of optimum amount of Fe_3_O_4_ nanospheres may enhance the electronic conductivity of the *t*-C_3_N_4_ and attribute the boosting of LPS adsorption.

**Fig. 3:**
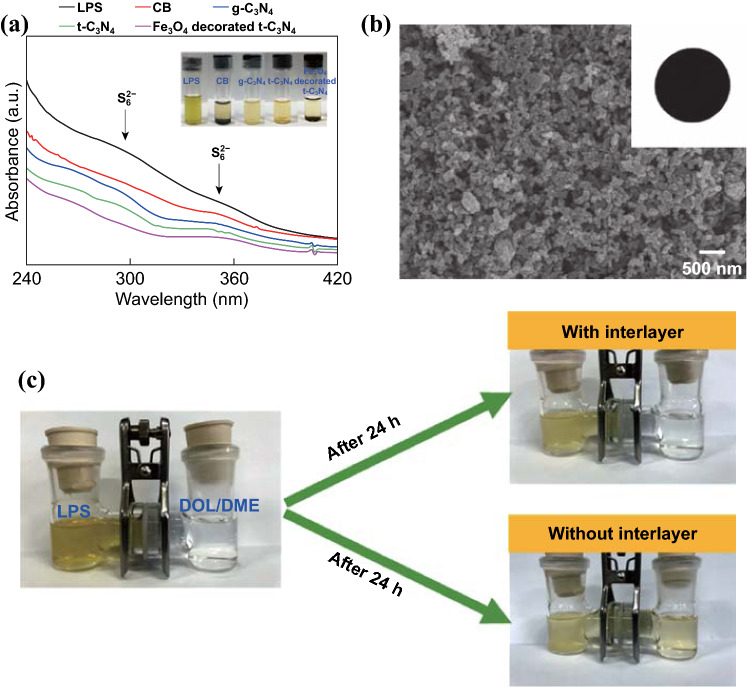
a UV–Vis spectra recorded for Li_2_S_6_, carbon black (CB), g-C_3_N_4_, *t*-C_3_N_4_, and Fe_3_O_4_/*t*-C_3_N_4_ as well as their corresponding optical images (inset). **b** SEM image of the surface of the interlayer constructed using the Fe_3_O_4_/*t*-C_3_N_4_. **c** Optical images obtained during the LPS adsorption test using an H-type cell with an Fe_3_O_4_/*t*-C_3_N_4_ interlayer and without interlayer

Figure 3a shows the adsorption capability toward LPS exhibited by the as-prepared materials evaluated by ultraviolet–visible (UV–Vis) spectroscopy using a 0.5 mmol L^−1^ Li_2_S_6_ DOL–DME solution containing 10 mg of the as-prepared materials [4, 26]. An absorption region of 240–420 nm can be observed in the LPS solution. The two characteristic peaks centered at 280 and 340 nm can be ascribed to the S_6_^2−^ species. After adding the four different materials (CB, g-C_3_N_4_, *t*-C_3_N_4_, and Fe_3_O_4_/*t*-C_3_N_4_) into the Li_2_S_6_ solution, the color of the solution was noticeably changed after 1 h from dark yellow to light yellow (Fig. 3a (inset)). Moreover, the peak intensities of the S_6_^2−^ species decreased significantly after adding the materials with the greatest decrease in the absorbance observed at 280 and 340 nm displayed by the Fe_3_O_4_/*t*-C_3_N_4_ [4, 26]. These results clearly demonstrate that Fe_3_O_4_/*t*-C_3_N_4_ can efficiently adsorb the LPS due to its high surface area and high affinity toward LPS.

Figure S6 shows the N_2_ adsorption isotherms obtained for g-C_3_N_4_, *t*-C_3_N_4_, and Fe_3_O_4_/*t*-C_3_N_4_. The Brunauer–Emmett–Teller (BET) specific surface area of the Fe_3_O_4_/*t*-C_3_N_4_ was estimated to be 71.27 m^2^ g^−1^ from the adsorption isotherm, which was much higher than those of g-C_3_N_4_ (10.86 m^2^ g^−1^) and *t*-C_3_N_4_ (33.71 m^2^ g^−1^). The large surface area of Fe_3_O_4_/*t*-C_3_N_4_ will lead to effective LPS regulation, facile electronic transfer, and remarkable battery performance.

### Fabrication of Fe_3_O_4_/*t*-C_3_N_4_ Interlayer and Evaluation of LPS Adsorption

An interlayer was prepared via a simple coating process using Fe_3_O_4_/*t*-C_3_N_4_, CB, and a polymer binder on a commercial Celgard separator. The surface morphology of the as-prepared interlayer was observed using SEM (Fig. 3b). Moreover, the cross-sectional morphology of the as-prepared interlayer is shown in Fig. S7. The thickness of the interlayer was controlled at ~ 10 μm; a thickness of ~ 10 μm is known to be appropriate without any significant disruption to ion transfer [27, 28].

Figure S8 shows that the interlayer based on Fe_3_O_4_/*t*-C_3_N_4_ presents stable mechanical properties upon folding. After five folding cycles, no damage was observed in the interlayer, such as cracks, tears, and exfoliation.

In agreement with the results obtained during the LPS adsorption study (Fig. 3a), the interlayer prepared using the Fe_3_O_4_/*t*-C_3_N_4_ also exhibits the efficient regulation of LPS in the H-type cell test (Fig. 3c). After 24 h, H-type cell with interlayer did not allow the movement of LPS through passing the as-prepared interlayer, which means the efficient suppression of LPS. However, in the H-type cell without using interlayer, the left and right side of the H-type cell turned to similar color (light yellow), which means penetrating the LPS and reaching equilibrium state of LPS concentration. These results demonstrate that the application of Fe_3_O_4_/*t*-C_3_N_4_ as an interlayer for Li–S batteries can attribute to enhanced battery performance.

### Electrochemical Characterization of Li–S Cell with Fe_3_O_4_/*t*-C_3_N_4_ Interlayer

**Fig. 4:**
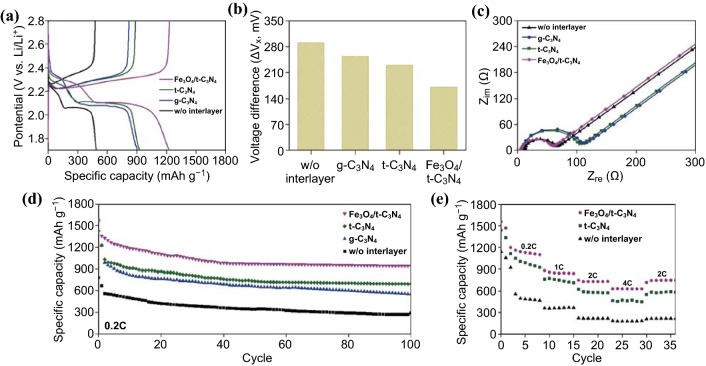
a Galvanostatic charge–discharge curves obtained for the as-prepared Li–S cells. **b** Voltage difference observed between the charge and discharge plateau obtained for the as-prepared Li–S cells. **c** EIS data obtained for the Li–S cells before cycling. **d** Discharging specific capacities of the Li–S cells observed at 0.2 C and **e** rate capability observed for the Li–S cells at 0.2, 1, 2, and 4 C

Figure 4 shows the effects of the as-prepared interlayer on the battery performance investigated using electrochemical analysis. Figure 4a presents the galvanostatic charge–discharge curves obtained at 0.2 C after activating at 0.05 C for two cycles. The Li–S cell constructed using the Fe_3_O_4_/*t*-C_3_N_4_ interlayer exhibits an enhanced specific capacity of 1245 mAh g^−1^, which indicates a 300 ~ 400% increase in capacity when compared to that of the Li–S cell constructed without an interlayer. Moreover, it shows a small voltage difference (ΔV_x_) between the charge and discharge plateaus (160 mV; Fig. 4b). A specific capacity of 901 mAh g^−1^ and ΔV_x_ value of 250 mV was exhibited by the Li–S cell constructed using the g-C_3_N_4_ interlayer. The Li–S cell prepared using the *t*-C_3_N_4_ interlayer displays a specific capacity of 971 mAh g^−1^ and ΔV_x_ value of 225 mV. The voltage difference observed between the charge and discharge plateaus shows the polarization and roundtrip energy efficiency of the cell. Lower polarization (lower voltage difference) represents a more kinetically efficient reaction process with a smaller barrier [29, 30]. These results are also consistent with the analysis through cyclic voltammograms in Fig. S9. Li–S cell with Fe_3_O_4_/*t*-C_3_N_4_ interlayer shows higher reduction peaks and a lower oxidation peak with large current density when compared to other two cells (with g-C_3_N_4_ interlayer and *t*-C_3_N_4_ interlayer). This suggests that the existence of the Fe_3_O_4_/*t*-C_3_N_4_ interlayer provides the efficient electrochemical redox process because of its increased electrical conductivity and strong adsorption for LPS [31, 32].

Figure 4c shows the electrochemical impedance spectroscopy (EIS) results obtained for the Li–S cells constructed using the various interlayers before cycling (see Figs. S10 and S11 for a detailed the EIS data and used EIS circuit). Before cycling, one depressed semicircle is observed, which denotes the charge transfer resistance (*R*_ct_) and characterizes the local redox reactions in which the active material obtains an electron from the conductive agent and captures a lithium ion from the liquid electrolyte [4, 25, 29]. Before cycling, the Li–S cells constructed with and without an interlayer using the Fe_3_O_4_/*t*-C_3_N_4_ exhibit lower *R*_ct_ values when compared to the Li–S cells prepared using the g-C_3_N_4_, and *t*-C_3_N_4_. For the Li–S cells prepared with an interlayer consisting of g-C_3_N_4_, and *t*-C_3_N_4_, the diffusion of Li ions may be restricted by physical barrier effect. In addition, the resistance increases slightly resulted from their relatively low electronic conductivity. However, there was no increase in the resistance observed for the Li–S cell prepared using the Fe_3_O_4_/*t*-C_3_N_4_ interlayer due to the improved electronic conductivity provided by Fe_3_O_4_ [33].

Figure 4d shows the results obtained for the battery performance tests performed using the as-prepared interlayers (g-C_3_N_4_, *t*-C_3_N_4_, and Fe_3_O_4_/*t*-C_3_N_4_) as well as a comparison of their performance with the Li–S battery prepared without an interlayer. The Li–S cell without using interlayer showed an initial specific capacity of 540 mAh g^−1^ at 0.2 C and a specific capacity of 262 mAh g^−1^ after 100 cycles. The Li–S cells prepared using an g-C_3_N_4_ and *t*-C_3_N_4_ interlayer exhibit further improved battery performance when compared to the Li–S cells constructed with and without an interlayer (for the g-C_3_N_4_ interlayer, the specific capacity was 603 mAh g^−1^ at 0.2 C after 100 cycles; the *t*-C_3_N_4_ interlayer shows a specific capacity of 810 mAh g^−1^ at 0.2 C after 100 cycles). As expected, g-C_3_N_4_ and *t*-C_3_N_4_ enhance the LPS adsorption capacity, which can improve the battery performance. Moreover, the Li–S cell prepared using the Fe_3_O_4_/*t*-C_3_N_4_ interlayer delivers a remarkable enhancement, such as an initial discharge capacity of 1255 mAh g^−1^ at 0.2 C and a retained discharging capacity of 1048 mAh g^−1^ after 100 cycles. In comparison with the controlled amount of Fe_3_O_4_/*t*-C_3_N_4_ interlayer (Fig. S12), the Li–S cell constructed using the Fe_3_O_4_/*t*-C_3_N_4_ interlayer shows a 400% improved battery performance compared with Li–S cell without using interlayer. Figure S10 shows the EIS analysis results obtained after 100 cycles. The Li–S cell prepared with the Fe_3_O_4_/*t*-C_3_N_4_ interlayer presents a smaller resistance (*R*_int_ and *R*_ct_, see the electronic supplementary material for details) when compared to those of the as-prepared Li–S cell. This was attributed to the activation of the sulfur cathode during cycling, and the data suggest that Fe_3_O_4_/*t*-C_3_N_4_ facilitates relatively fast electronic transfer and provides an efficient electrochemical system for suppressing the LPS shuttle effect, which leads to its high battery performance. Moreover, after cycling, the surface of interlayer did not present crack and collapse of interlayer materials (Fig. S13).

The fabricated Li–S cells were evaluated in terms of their rate capabilities with current densities ranging from 0.05 to 4 C to further analyze the battery performance, as shown in Fig. 4e. The average discharge capacities obtained for the Li–S cell constructed using a Fe_3_O_4_/*t*-C_3_N_4_ interlayer show highly stable rate capabilities comparable to those demonstrated by the Li–S cells prepared with and without an interlayer prepared using *t*-C_3_N_4_. Moreover, the Li–S cell constructed using a Fe_3_O_4_/*t*-C_3_N_4_ interlayer delivers a desirable battery performance at a high C-rate (830 mAh g^−1^ at 2 C and 680 mAh g^−1^ at 4 C). The high regulation of the LPS and enhanced electronic conductivity of the as-prepared interlayer result in a high performance of the Li–S battery at a high C-rate.

**Fig. 5:**
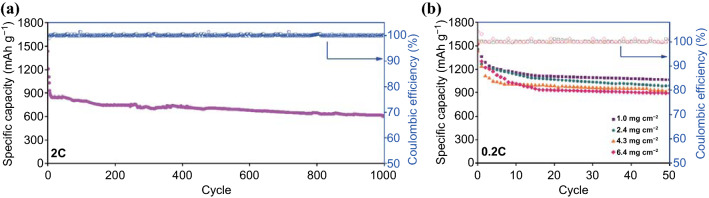
a Discharge specific capacities obtained for the Li–S cell prepared with an Fe_3_O_4_-decorated *t*-C_3_N_4_ interlayer operated at 2 C during the stability test and **b** discharge specific capacity of the high-sulfur-loading cathode Fe_3_O_4_/*t*-C_3_N_4_ interlayer operated at 0.2 C

For commercial applications, the loading density of sulfur and the C-rate are important factors [4, 30]. Figure 5a shows the results of the long-term cycling test obtained for the Li–S cell prepared with the Fe_3_O_4_/*t*-C_3_N_4_ interlayer operated at 2 C. The Li–S cell with the Fe_3_O_4_/*t*-C_3_N_4_ interlayer exhibited an initial discharge capacity of 828 mAh g^−1^ at 2 C, at which most portable batteries are rated, and its capacity remained at 658 mAh g^−1^ over 1000 cycles (a capacity retention decay of 0.02% per cycle). The long-term cycling test results demonstrate the remarkable effects of regulating the LPS and the enhanced electronic properties of the Fe_3_O_4_/*t*-C_3_N_4_ interlayer.

Furthermore, an increased sulfur loading is important for the commercial application of LSBs. For example, the batteries used in electric vehicles (EVs) require a sulfur loading of > 2.0 mg cm^−2^ for a specific energy density of > 400 Wh kg^−1^ [4, 34]. Thus, the specific capacity of the Li–S cell prepared with the Fe_3_O_4_/*t*-C_3_N_4_ interlayer was evaluated as a function of the sulfur loading (1.0, 2.4, 4.3, and 6.4 mg cm^−2^) at 0.2 C. Figure 5b shows that the Li–S cell maintained its stability upon increasing the sulfur loading and its Coulombic efficiency reached ~ 99%. The efficiently regulated LPS and enhanced electronic properties of Fe_3_O_4_/*t*-C_3_N_4_ result in long-term cycling stability even with a high-sulfur loading.

Table S3 shows a comparison of the battery performance observed for the Li–S cells prepared using various N-doped carbonaceous-based interlayers. Among the interlayers studied, the Fe_3_O_4_/*t*-C_3_N_4_ interlayer induces the highest cycling stability with a capacity decay of 0.02% per cycle at a high C-rate (2 C). Therefore, based on these results, employing a Fe_3_O_4_/*t*-C_3_N_4_ interlayer in an LSB will facilitate efficient battery usage and result in impressive battery performance.

## Conclusions

We have explored the hierarchical Fe_3_O_4_/*t*-C_3_N_4_ nanostructures as an interlayer for advanced LSBs. *t*-C_3_N_4_ has a high affinity toward LPS and increased surface area, and the embedded Fe_3_O_4_ nanospheres on the surface of *t*-C_3_N_4_ enhance the electrical conductivity and LPS anchoring properties. The synergistic effect between the *t*-C_3_N_4_ and embedded Fe_3_O_4_ nanospheres exhibits a remarkable battery performance with a low capacity decay per cycle of 0.02% at 2 C over 1000 cycles as well as stable cycling at 6.4 mg cm^−2^ for a high-sulfur-loading cathode. The Fe_3_O_4_/*t*-C_3_N_4_ interlayer can be employed in various energy storage devices as well as advanced LSBs.

## Electronic supplementary material

Below is the link to the electronic supplementary material.

Supplementary material 1 (PDF 957 kb)
