# Supplementary material for: Strongly Anchoring Polysulfides by Hierarchical Fe3O4/C3N4 Nanostructures for Advanced Lithium–Sulfur Batteries
Source: Nanomicro Lett. 2020 Jul 1;12:139. doi: 10.1007/s40820-020-00475-5 (PMC7770789; doi:10.1007/s40820-020-00475-5)
Supplement: Supplementary file 1 — Supplementary material 1 (PDF 957 kb) [file 40820_2020_475_MOESM1_ESM.pdf]

Supporting Information for

Strongly Anchoring Polysulfides by Hierarchical Fe_3O_4/C_3N_4 Nanostructures for Advanced Lithium-Sulfur Batteries

Soochan Kim[1], Simindokht Shirvani-Arani[2], Sungsik Choi[1], Misuk Cho[1], Youngkwan Lee[1, *]

[1]School of Chemical Engineering, Sungkyunkwan University, Suwon 16419, Republic of Korea

[2] Nuclear Science and Technology Research Institute (NSTRI), Tehran, 14395-834, Iran

*Corresponding author. E-mail: yklee@skku.edu (Youngkwan Lee)

S1 Experimental Detail and Mechanism

S1.1 Synthesis of Sulfur/carbon Black (CB) Composites

To synthesize the S/CB composite, sulfur and CB were mixed at a mass ratio of 7:3. The mixture was then heated at 155 °C for 10 h under Ar atmosphere, in order to melt sulfur into the pores in carbon matrix. (The detailed sulfur content in Table S1)

S1.2 Rolling Mechanism of Tube C_3N_4

The formation process of C_3N_4 nanotube could follow a rolling-up mechanism [S1-S3]. During the calcination process, a large amount of NH_3 gas was released from the pyrolysis of melamine. The released NH_3 gas went vertically through the moderately packed melamine layers to form slightly rolled sheet like g-C_3N_4. Formed sheet like g-C_3N_4 tended to further roll into tubular structures to minimize the total surface free energy [S3, S4].

S1.3 Formation of Fe_3O_4 Nanosphere Decorated C_3N_4 Nanotube

Fe_3O_4 nanospheres are prepared by the nucleation and growth of the nuclei [S5, S6]. A short single burst of nucleation occurs when the concentration of constituent species reaches critical supersaturation. Then, the nuclei so obtained are allowed to grow uniformly by diffusion of solutes from the solution to their surface of C_3N_4 nanotube until the final size is attained. In the high concentration of Fe_3O_4 precursor solution, the aggregation of Fe_3O_4 on the surface of C_3N_4 nanotube could be formed.

S2 Supplementary Figures and Tables

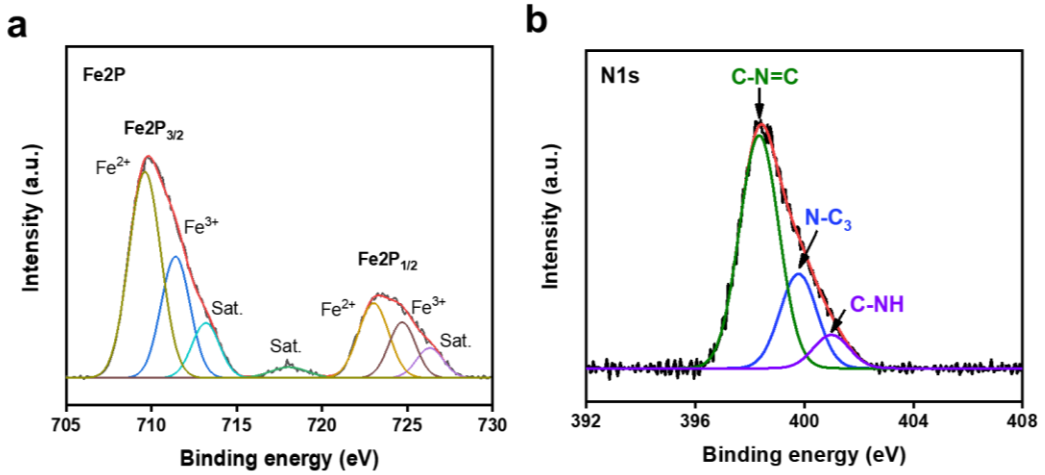

Fig. S1 Detailed XPS peaks Fe 2P (**a**) and N1s (**b**) of Fe_3O_4/t-C_3N_4

In N1S XPS spectra, 398.9, 400.3, and 401.4 eV corresponding well to sp^2-hybridized nitrogen (C-N=C), sp^3-hybridized nitrogen (N-C3) and amino C-NH group are also found. The peak at 398.9 eV is considered to be the dominative part in g-C_3N_4 [S7].

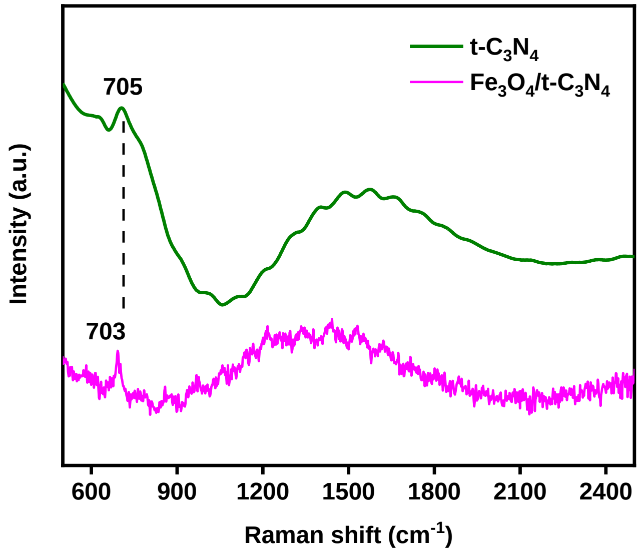

Fig. S2 Raman spectra of t-C_3N_4 and Fe_3O_4/t-C_3N_4

Figure S2 presents the Raman spectra of t-C_3N_4 and Fe_3O_4/t-C_3N_4 at 785 nm. In spectra of t-C_3N_4, there is a signature peak of C_3N_4 at 705 cm^{-1} from the vibration modes of CN heterocycles appear [S8]. The Raman spectra of Fe_3O_4/t-C_3N_4 showed down-shifted peak of 703 cm^{-1} which might be due to the polar interaction of Fe_3O_4 and t-C_3N_4 [S8, S9].

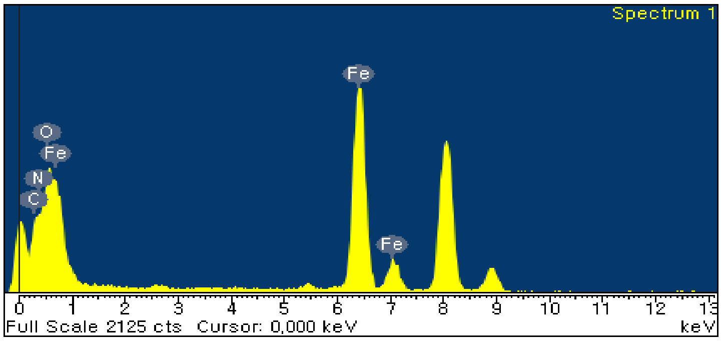

Fig. S3 EDS spectrum of Fe_3O_4/t-C_3N_4

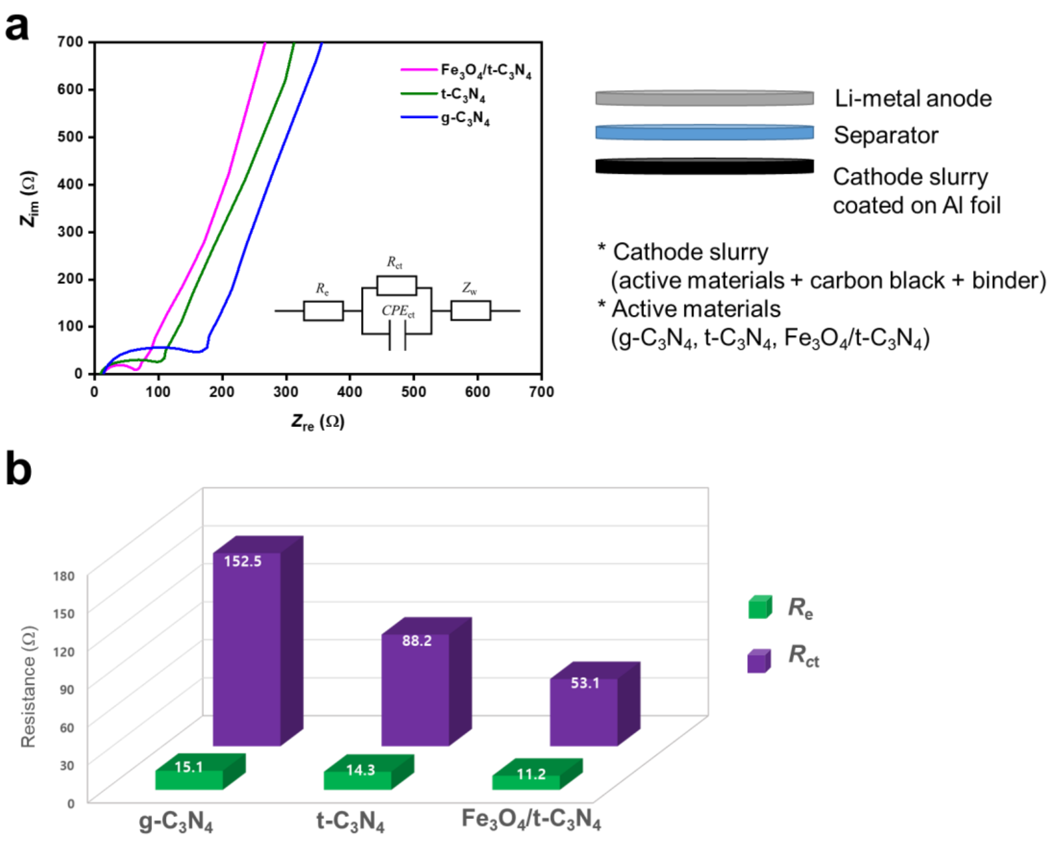

Fig. S4 (**a**) EIS data for confirmation of electronic conductivity and (**b**) detailed results of Fig. S3a

R_e is electrolyte resistance and R_{ct} (one depressed semicircle) is the charge transfer resistance which is related with conductivity of active materials.

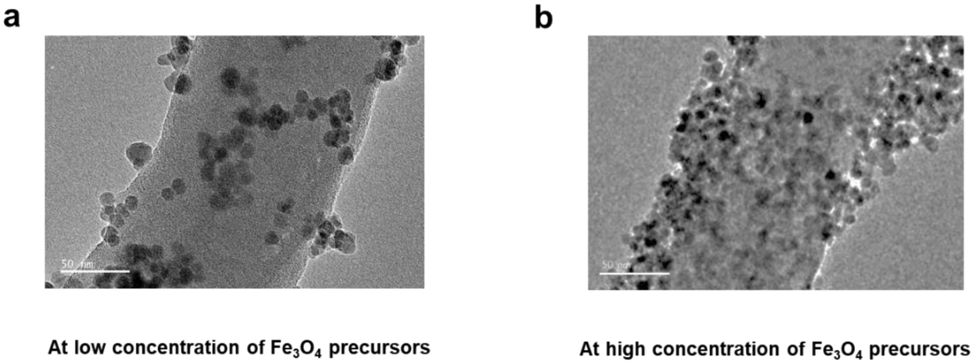

Fig. S5 TEM images of Fe_3O_4/t-C_3N_4 according to the amounts of Fe_3O_4 (Scale bar: 50 nm)

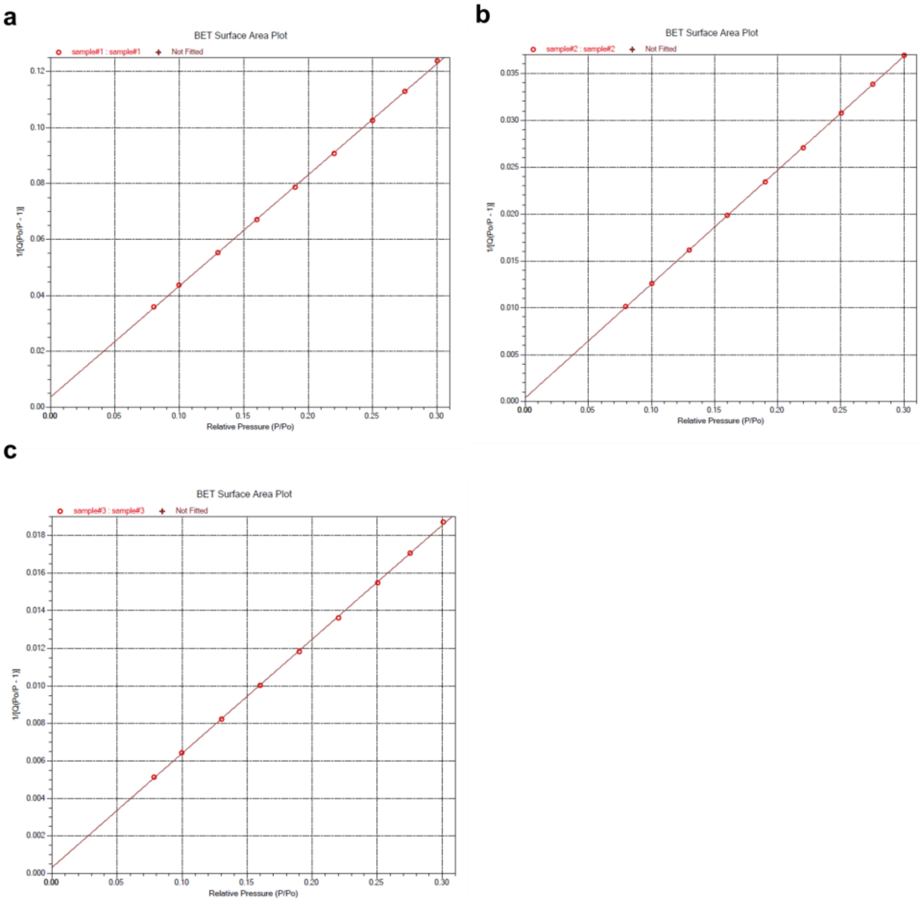

Fig. S6 BET surface area plots which obtained by N_2 adsorption isotherm analysis. (**a**) g- C_3N_4, (**b**) t-C_3N_4, and (**c**) Fe_3O_4/t-C_3N_4

P/P_o: Relative Pressure

Q: Quantity Adsorbed (cm^3/g, STP)

$1/[Q(P_o/P-1)]$; Ratio of relative saturation ($= P/(P_o-P)$) to adsorbed gas volume per gram of solid

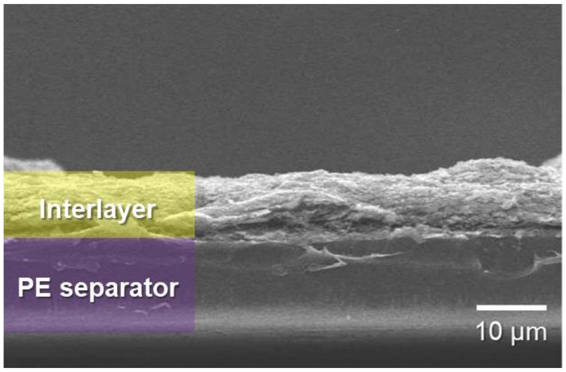

Fig. S7 Cross-sectional SEM image of Fe_3O_4/t-C_3N_4 interlayer (Scale bar: 10 μm)

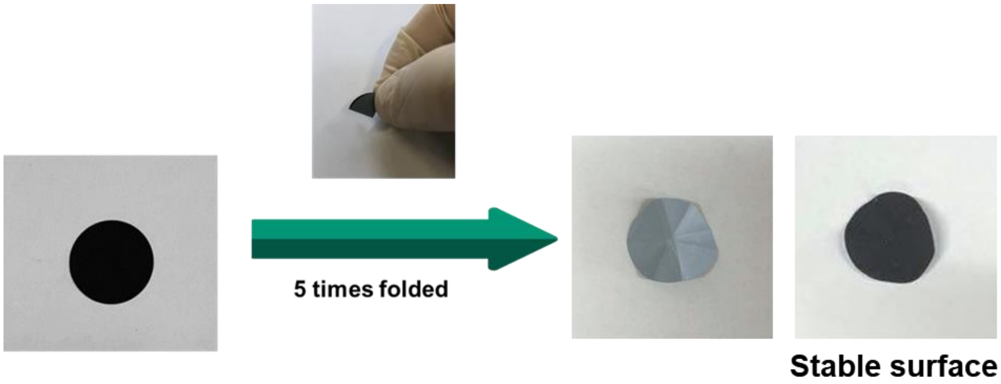

Fig. S8 Optical images of folding test of separator modified with Fe_3O_4/t-C_3N_4 interlayer

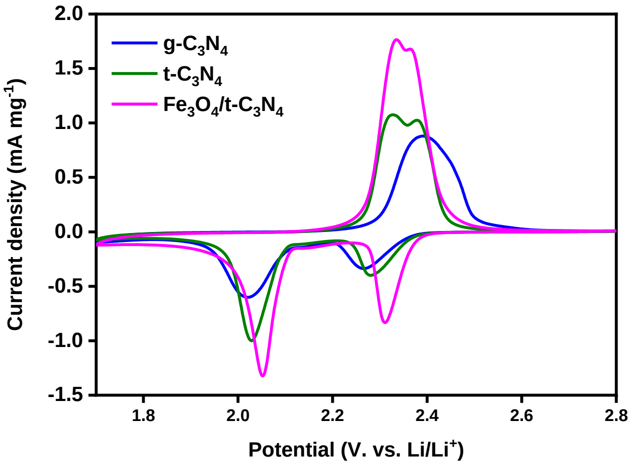

Fig. S9 Cyclic voltammograms of Li-S cells with prepared interlayers after 10 cycles with a scan rate of 0.1 mVs^{-1}

Figure S9 shows the cyclic voltammograms (CV) after 10 cycles from 1.7 to 2.8 V vs. Li/Li^+ with a scan rate of 0.1 mV s^{-1}.

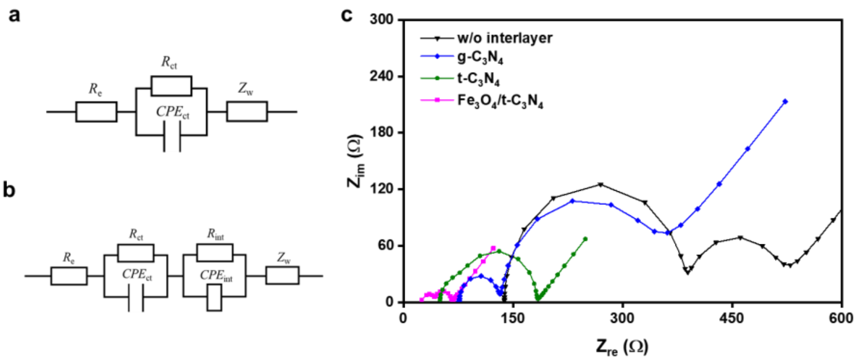

Fig. S10 EIS circuits (**a, b**) and EIS data of Li-S cell after cycling (**c**)

After cycling, Li-S cells showed two semicircles where the semicircle in the high-to-middle frequency region (right) is due to the charge-transfer resistance (R_{ct}), while the semicircle in the high frequency region (left) can be ascribed to the interfacial contact resistance (R_{int}) between the electrolyte and the cathode [S10].

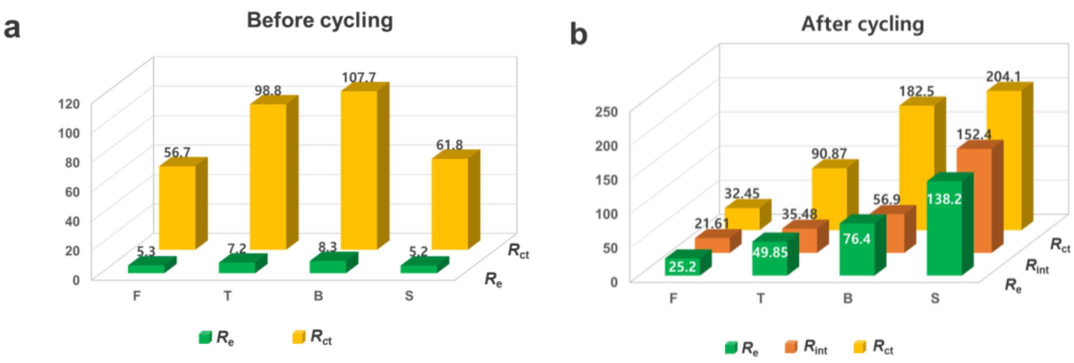

Fig. S11 Detailed EIS results about Li-S cell before (**a**) and after cycling (**b**)

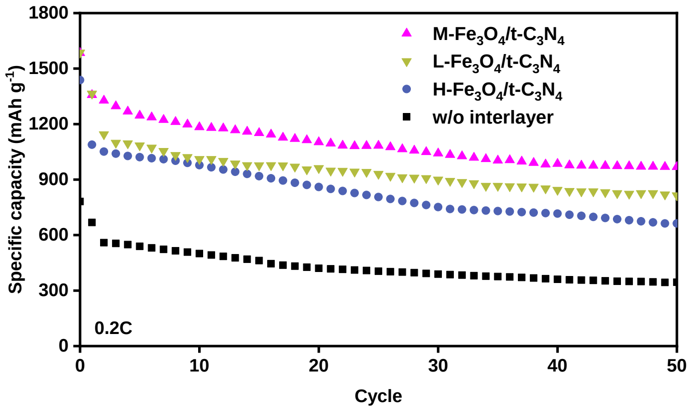

Fig. S12 Battery performance of Li-S cell with Fe_3O_4/t-C_3N_4 interlayer according to the amount of Fe_3O_4

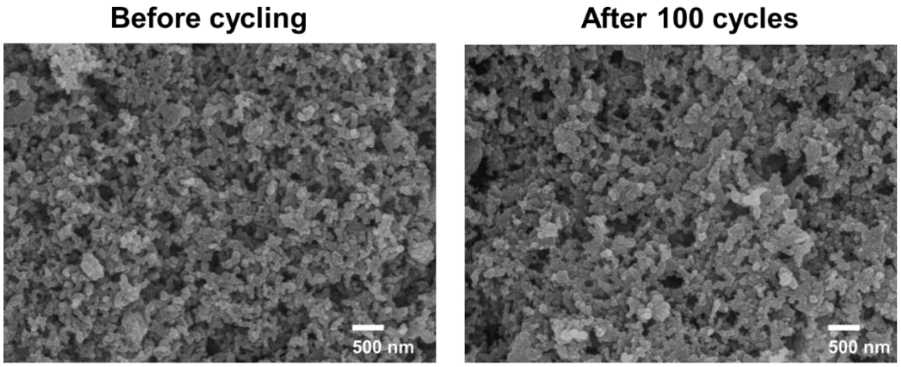

Fig. S13 SEM images of the surface of Fe_3O_4/t-C_3N_4 interlayer before and after cycling

Table S1 Elemental analysis of S/carbon black(CB) composite

**Elemental composition (wt. %)**	**Sample**
	S/CB composite
Carbon	29.3 (±1.2)
Hydrogen	0.5 (±1.0)
Sulfur	70.1 (±1.0)

The theoretical value of S and CB ratio is 7:3 (w/w). The S/C ratios in the cases of prepared sample is a similar result of theoretical value.

Table S2 Elemental analysis of prepared materials (g-C_3N_4 and t-C_3N_4)

**Elemental composition (wt%)**	**Sample**	
	**g-C_3N_4**	**t-C_3N_4**
Carbon	33.5 (±1.2)	34.5 (±1.0)
Hydrogen	1.3 (±1.0)	1.6 (±0.8)
Nitrogen	58.7 (±2.0)	57.6 (±1.0)

The theoretical value of C, N atomic ratio is 0.75. The C, N ratios in the cases of g-C_3N_4 and t-C_3N_4 are found to be a similar result of theoretical value (0.66 and 0.69, respectively).

Table S3 Comparison about battery performance of Li-S batteries with various N-doped carbon- and carbon nitride-based interlayer

Materials for interlayer	N contents (wt. %)	Capacity after cycling (mAh g^{-1})	Cycle	Rate	Capacity retention decay per cycle (%)	References
N-doped porous carbon	9.50	689	200	0.5 C	0.210	[S11]
N-doped graphene	10.46	956	50	0.1 C	0.710	[S12]
Carbonized Polyacrylonitrile fiber	unknown	710	200	0.3 C	0.127	[S13]
N-doped graphene	2.68	666.8	300	0.5 C	0.037	[S14]
N-doped carbon from newspaper	unknown	504	200	0.5 C	0.260	[S15]
g-C_3N_4 paper	33.87	1,271.5	400	0.1 C	0.068	[S16]
Reduced graphene oxide/g-C_3N_4	47.9	~500	800	1 C	0.056	[S17]
MoS_2/N-doped carbontube	9.2	896.7	200	0.2 A g^{-1}	0.100	[S18]
ZrO_2/N-doped carbon nanofiber	9.7	759	500	0.2 C	0.039	[S19]
N, S-doped porous carbon	3.47	609	300	0.5 C	0.060	[S20]
C_3N_4 phosphorus	unknown	850	700	0.5 C	0.041	[S21]
Graphene/g-C_3N_4	unknown	612.4	1,000	1 C	0.048	[S22]
N, B-doped carbon nanofiber	unknown	443	1,000	1 C	0.058	[S23]
N, O-doped carbon nanofiber	3.5	420	1,000	1 C	0.040	[S24]
Hierarchical nanostructured C_3N_4 embedded with Fe_3O_4	**40.32**	**658**	**1,000**	**2 C**	**0.020**	**Our work**

Supplementary References

[S1] Chai G, Lin C, Zhang M, Wang J, Cheng W. First-principles study of CN carbon nitride nanotubes. Nanotechnology. **21**(19), 195702 (2010). http://doi.org/10.1088/0957-4484/21/19/195702

[S2] Gao J, Zhou Y, Li Z, Yan S, Wang N, Zou Z. High-yield synthesis of millimetre-long, semiconducting carbon nitride nanotubes with intense photoluminescence emission and reproducible photoconductivity. Nanoscale **4**(12), 3687-3692 (2012). http://doi.org/10.1039/C2NR30777D

[S3] Pan C, Xu J, Wang Y, Li D, Zhu Y. Dramatic activity of C_3N_4/bipo4 photocatalyst with core/shell structure formed by self-assembly. Adv. Funct. Mater. **22**(7), 1518-1524 (2012). http://doi.org/10.1002/adfm.201102306

[S4] Wang S, Li C, Wang T, Zhang P, Li A, Gong J. Controllable synthesis of nanotube-type graphitic c3n4 and their visible-light photocatalytic and fluorescent properties. J. Mater. Chem. A **2**(9), 2885-2890 (2014). http://doi.org/10.1039/C3TA14576J

[S5] LaMer VK, Dinegar RH. Theory, production and mechanism of formation of monodispersed hydrosols. J. Am. Chem. Soc. **72**(11), 4847-4854 (1950). http://doi.org/10.1021/ja01167a001

[S6] Tartaj P, Morales MadP, Veintemillas-Verdaguer S, Gonz lez-Carre o T, Serna CJ. The preparation of magnetic nanoparticles for applications in biomedicine. J. Phys. D Appl. Phys. **36**(13), R182-R197 (2003). http://doi.org/10.1088/0022-3727/36/13/202

[S7] Ji C, Yin S-N, Sun S, Yang S. An in situ mediator-free route to fabricate Cu_2O/g-C_3N_4 type-ii heterojunctions for enhanced visible-light photocatalytic h2 generation. Appl. Surf. Sci. **434**, 1224-1231 (2018). http://doi.org/10.1016/j.apsusc.2017.11.233

[S8] Zhu Z, Lu Z, Wang D, Tang X, Yan Y, Shi W, Wang Y, Gao N, Yao X, Dong H. Construction of high-dispersed Ag/Fe_3O_4/g-C_3N_4 photocatalyst by selective photo-deposition and improved photocatalytic activity. Appl. Catal. B **182**, 115-122 (2016). doi.org/10.1016/j.apcatb.2015.09.029

[S9] Y. P. Yew, K. Shameli, M. Miyake, N.B.B. A. Khairudin, S.E.B. Mohamad, H. Hara, M.F.B.M. Nordin, K.X. Lee. An eco-friendly means of biosynthesis of superparamagnetic magnetite nanoparticles via marine polymer. IEEE Trans. Nanotechnol. **16**(6), 1047-1052 (2017). doi.org/10.1109/TNANO.2017.2747088

[S10] Kim S, Cho M, Lee Y. Multifunctional chitosan–rGO network binder for enhancing the cycle stability of li–s batteries. Adv. Funct. Mater. **30**(10), 1907680 (2020). http://doi.org/10.1002/adfm.201907680

[S11] Cao Z, Zhang J, Ding Y, Li Y, Shi M, Yue H, Qiao Y, Yin Y, Yang S. In situ synthesis of flexible elastic n-doped carbon foam as a carbon current collector and interlayer for high-performance lithium sulfur batteries. J. Mater. Chem. A **4**(22), 8636-8644 (2016). http://doi.org/10.1039/C6TA01855F

[S12] Deng H, Yao L, Huang Q-A, Su Q, Zhang J, Du G. Highly improved electrochemical performance of li-s batteries with heavily nitrogen-doped three-dimensional porous graphene interlayers. Mater. Res. Bull. **84**, 218-224 (2016). http://doi.org/10.1016/j.materresbull.2016.08.014

[S13] Li Q, Liu M, Qin X, Wu J, Han W, Liang G, Zhou D, He Y-B, Li B, Kang F. Cyclized-polyacrylonitrile modified carbon nanofiber interlayers enabling strong trapping of polysulfides in lithium–sulfur batteries. J. Mater. Chem. A **4**(33), 12973-12980 (2016). http://doi.org/10.1039/C6TA03918A

[S14] Wu H, Huang Y, Xu S, Zhang W, Wang K, Zong M. Fabricating three-dimensional hierarchical porous n-doped graphene by a tunable assembly method for interlayer assisted

lithium-sulfur batteries. Chem. Eng. J. **327**(855-867 (2017). http://doi.org/10.1016/j.cej.2017.06.164

[S15] Chang C-H, Chung S-H, Manthiram A. Transforming waste newspapers into nitrogen-doped conducting interlayers for advanced li–s batteries. Sustain. Energy Fuels **1**(3), 444-449 (2017). http://doi.org/10.1039/C7SE00014F

[S16] Wutthiprom J, Phattharasupakun N, Khuntilo J, Maihom T, Limtrakul J, Sawangphruk M. Collaborative design of Li–S batteries using 3d n-doped graphene aerogel as a sulfur host and graphitic carbon nitride paper as an interlayer. Sustain. Energy Fuels **1**(8), 1759-1765 (2017). http://doi.org/10.1039/C7SE00291B

[S17] Wutthiprom J, Phattharasupakun N, Sawangphruk M. Designing an interlayer of reduced graphene oxide aerogel and nitrogen-rich graphitic carbon nitride by a layer-by-layer coating for high-performance lithium sulfur batteries. Carbon **139,** 945-953 (2018). http://doi.org/10.1016/j.carbon.2018.08.008

[S18] Zhao X, Wang G, Liu X, Zheng X, Wang H. Ultrathin MoS_2 with expanded interlayers supported on hierarchical polypyrrole-derived amorphous n-doped carbon tubular structures for high-performance li/na-ion batteries. Nano Res. **11**(7), 3603-3618 (2018). http://doi.org/10.1007/s12274-017-1927-2

[S19] Li Y, Zhu J, Shi R, Dirican M, Zhu P, Yan C, Jia H, Zang J, He J, Zhang X. Ultrafine and polar ZrO_2-inlaid porous nitrogen-doped carbon nanofiber as efficient polysulfide absorbent for high-performance lithium-sulfur batteries with long lifespan. Chem. Eng. J. **349**, 376-387 (2018). http://doi.org/10.1016/j.cej.2018.05.074

[S20] Jiang S, Chen M, Wang X, Zhang Y, Huang C, Zhang Y, Wang Y. Honeycomb-like nitrogen and sulfur dual-doped hierarchical porous biomass carbon bifunctional interlayer for advanced lithium-sulfur batteries. Chem. Eng. J. **355**, 478-486 (2019). http://doi.org/10.1016/j.cej.2018.08.170

[S21] Do V, Deepika, Kim MS, Kim MS, Lee KR, Cho WI. Carbon nitride phosphorus as an effective lithium polysulfide adsorbent for lithium–sulfur batteries. ACS Appl. Mater. Interfaces **11**(12), 11431-11441 (2019). http://doi.org/10.1021/acsami.8b22249

[S22] Qu L, Liu P, Yi Y, Wang T, Yang P, Tian X, Li M, Yang B, Dai S. Enhanced cycling performance for lithium–sulfur batteries by a laminated 2d g-c3n4/graphene cathode interlayer. ChemSusChem. **12**(1), 213-223 (2019). http://doi.org/10.1002/cssc.201802449

[S23] Zhu J, Pitcheri R, Kang T, Jiao C, Guo Y, Li J, Qiu Y. A polysulfide-trapping interlayer constructed by boron and nitrogen co-doped carbon nanofibers for long-life lithium sulfur batteries. J. Electroanal. Chem **833**, 151-159 (2019). http://doi.org/10.1016/j.jelechem.2018.11.010

[S24] Gao T, Yu Z, Huang Z-H, Yang Y. Nitrogen/oxygen dual-doped carbon nanofibers as an electrocatalytic interlayer for a high sulfur content lithium–sulfur battery. ACS Appl. Energy Mater. **2**(1), 777-787 (2019). http://doi.org/10.1021/acsaem.8b01840
